# Supplementary material for: Identification of Host PDZ-Based Interactions with the SARS-CoV-2 E Protein in Human Monocytes
Source: Int J Mol Sci. 2023 Aug 14;24(16):12793. doi: 10.3390/ijms241612793 (PMC10454406; doi:10.3390/ijms241612793)
Supplement: Supplementary file 1 [file ijms-24-12793-s001.zip › ijms-2510698-supplementary.pdf]

Avila-Flores et al., Supplementary material

Supplemental Table 1 Protein interactors classified by biological process

Leukocyte degranulation	Myeloid cell activation in immune response	Immune response	Neutrophil activation involved in immune response	Neutrophil degranulation	Exocytosis	Secretion	Protein targeting to membrane	Cotranslational protein targeting to membrane	Establishment of protein localization to endoplasmic reticulum	Translational initiation	Viral process	Viral transcription
		ANKHD1								ABCF1	C1QBP	
ANO6	ANO6	ANO6	ANO6	ANO6	ANO6	ANO6				EIF1	CALCOCO2	
		ATP6V0A2								EIF2B3	CD55	
		BCL2								EIF3M	CDK5RAP3	
BST1	BST1	BST1	BST1	BST1	BST1	BST1				EIF4E2	CHMP1A	
		C17orf62								EIF4G3	DHX58	
		C1QBP					EXOC4				DOCK2	
		CALCOCO2					GOLGA7				EIF3M	
CAND1	CAND1	CAND1	CAND1	CAND1	CAND1	CAND1				LARP1	ELMO1	
		CCR1			CCR1	CCR1			RAB3GAP1		F11R	
					CD109	CD109	RABGEF1				HCK	
CD44	CD44	CD44	CD44	CD44	CD44	CD44	RPL10	RPL10	RPL10	RPL10	IL16	
CD55	CD55	CD55	CD55	CD55	CD55	CD55	RPL10A	RPL10A	RPL10A	RPL10A	ITCH	
CSNK2B	CSNK2B	CSNK2B	CSNK2B	CSNK2B	CSNK2B	CSNK2B	RPL18A	RPL18A	RPL18A	RPL18A	ITGAL	
CSTB	CSTB	CSTB	CSTB	CSTB	CSTB	CSTB	RPL28	RPL28	RPL28	RPL28	KPNA2	
CYFIP1	CYFIP1	CYFIP1	CYFIP1	CYFIP1	CYFIP1	CYFIP1	RPL39	RPL39	RPL39	RPL39	LYN	
		DHX58					RPL4	RPL4	RPL4	RPL4	MOV10	
DIAPH1	DIAPH1	DIAPH1	DIAPH1	DIAPH1	DIAPH1	DIAPH1	RPL9	RPL9	RPL9	RPL9	NUP62	NUP62
DOCK2	DOCK2	DOCK2	DOCK2	DOCK2	DOCK2	DOCK2	RPLP1	RPLP1	RPLP1	RPLP1	NUP98	NUP98
		EXOSC3			EXOC4	EXOC4	RPS15	RPS15	RPS15	RPS15	PHB2	
						GNAS	RPS24	RPS24	RPS24	RPS24	PI4KA	
GOLGA7	GOLGA7	GOLGA7	GOLGA7	GOLGA7	GOLGA7	GOLGA7	RPS29	RPS29	RPS29	RPS29	PML	
HCK		HCK			HCK	HCK	RPS3A	RPS3A	RPS3A	RPS3A	PTBP1	
HVCN1	HVCN1	HVCN1	HVCN1	HVCN1	HVCN1	HVCN1	RPS4X	RPS4X	RPS4X	RPS4X	RAE1	RAE1
		IFI44L					RPS8	RPS8	RPS8	RPS8	RPL10	RPL10
IGF2R	IGF2R	IGF2R	IGF2R	IGF2R	IGF2R	IGF2R	SDCBP				RPL10A	RPL10A
		IL16					SEC61A1	SEC61A1	SEC61A1		RPL18A	RPL18A
		ITCH					SEC63	SEC63	SEC63		RPL28	RPL28
ITGAL	ITGAL	ITGAL	ITGAL	ITGAL	ITGAL	ITGAL	SRPR	SRPR			RPL39	RPL39
LAMTOR1	LAMTOR1	LAMTOR1	LAMTOR1	LAMTOR1	LAMTOR1	LAMTOR1	SSR1	SSR1			RPL4	RPL4
					LIN7A	LIN7A	22	18	18	21	RPL9	RPL9
LYN	LYN	LYN			LYN	LYN					RPLP1	RPLP1
						M6PR					RPS15	RPS15
MAGT1	MAGT1	MAGT1	MAGT1	MAGT1	MAGT1	MAGT1					RPS24	RPS24
		MEF2C									RPS29	RPS29
		MEFV									RPS3A	RPS3A
MGST1	MGST1	MGST1	MGST1	MGST1	MGST1	MGST1					RPS4X	RPS4X
		MYD88			MYO5A	MYO5A					RPS8	RPS8
NCKAP1L	NCKAP1L	NCKAP1L	NCKAP1L	NCKAP1L	NCKAP1L	NCKAP1L					SCRIB	
		NONO									SF3B2	
		PARP9									SND1	
		PML									SSRP1	
						PREB					STAT2	
PSMA5	PSMA5	PSMA5	PSMA5	PSMA5	PSMA5	PSMA5					STAU1	
PSMD1	PSMD1	PSMD1	PSMD1	PSMD1	PSMD1	PSMD1					SYK	
					RAB11FIP1	RAB11FIP1					TAP2	
RAP2B	RAP2B	RAP2B	RAP2B	RAP2B	RAP2B	RAP2B					TFRC	
		RBM14			SCIN	SCIN					TRIM22	
		RPL39			SCRIB	SCRIB					USP15	
SDCBP	SDCBP	SDCBP	SDCBP	SDCBP	SDCBP	SDCBP					USP7	
		SEC61A1									VAMP8	
		SFPQ				SLC29A1					VTA1	
SLC2A5	SLC2A5	SLC2A5	SLC2A5	SLC2A5	SLC2A5	SLC2A5					XPO5	
		STAT2									53	17
		STAT6										
STOM	STOM	STOM	STOM	STOM	STOM	STOM						
					STX11	STX11						
STXBP2	STXBP2	STXBP2	STXBP2	STXBP2	STXBP2	STXBP2						
STXBP3	STXBP3	STXBP3	STXBP3	STXBP3	STXBP3	STXBP3						
SURF4	SURF4	SURF4	SURF4	SURF4	SURF4	SURF4						
	SYK	SYK	SYK		SYK	SYK						
		TAP2										
TCIRG1	TCIRG1	TCIRG1	TCIRG1	TCIRG1	TCIRG1	TCIRG1						
					TMEM167A	TMEM167A						
TMEM30A	TMEM30A	TMEM30A	TMEM30A	TMEM30A	TMEM30A	TMEM30A						
TMEM63A	TMEM63A	TMEM63A	TMEM63A	TMEM63A	TMEM63A	TMEM63A						
TOLLIP	TOLLIP	TOLLIP	TOLLIP	TOLLIP	TOLLIP	TOLLIP						
		TRIM22										
VAMP8	VAMP8	VAMP8	VAMP8	VAMP8	VAMP8	VAMP8						
VAT1	VAT1	VAT1	VAT1	VAT1	VAT1	VAT1						
35	35	62	34	33	47	51						

Supplemental Table S1. Classification of GFP-SARS-CoV-2 E protein interactors according to GoTERM categories of biological process . Proteins classified into the distinct GoTERM categories are listed; the total number of proteins is indicated at the bottom. Color assigned to the different GoTERM categories as shown in Figure 3A.

Supplemental Table 2 Interactors classified by cellular region

Cell junction	Synapse	Endomembrane system	Endoplasmic reticulum membrane	Cytoplasmic vesicle	Cytoplasmic vesicle membrane	Secretory vesicle	Secretory granule membrane	Extracellular exosome	Specific granule	Cytosolic ribosome	Ribosome	Ribonucleoprotein complex
ADD1	ADD1	ABCF1						ABHD14B			ABCF1	ABCF1
ASAP1	ASAP1	ACSL3		AFTPH							NUFIP2	DHX15
ATP2B1	ATP2B1	AFTPH		AGFG1	AFTPH					RPL10	RPL10	DRG1
ATP6V0D1	ATP6V0D1	AGFG1		AGTRAP	AGTRAP					RPL10A	RPL10A	EIF1
C1QBP	C1QBP		ACSL3	ANO6	ANO6	ANO6	ANO6	ANO6	ANO6	RPL18A	RPL18A	EIF3M
CD44		AGTRAP	AGTRAP	ANP32E				ATP2B1		RPL28	RPL28	GPKOW
CORO2B		ANO6		ARAP1						RPL39	RPL39	HNRNPA0
CTNND1	CTNND1	ARAP1		ATP6AP1				ATP6AP1		RPL4	RPL4	HNRNPDL
CYFIP1	CYFIP1	ATP6AP1	ATP6AP1	ATP6V0A2	ATP6V0A2	ATP6V0A2				RPL9	RPL9	HNRNPL
DAGLB	DAGLB	ATP6V0A2		ATP6V0D1	ATP6V0D1	ATP6V0D1		ATP6V0D1		RPLP1	RPLP1	NCBP1
DCTN4		ATP6V0D1		ATP6V1C1				ATP6V1C1		RPS15	RPS15	NOB1
DENND1A	DENND1A	ATP6V1E1		ATP6V1E1				ATP6V1E1		RPS24	RPS24	NUFIP2
EXOC4	EXOC4	B3GAT3						B3GAT3		RPS29	RPS29	NUP62
F11R		BCL2	BCL2					BLMH		RPS3A	RPS3A	NUP98
FHOD1		BST1		BST1	BST1	BST1	BST1	BST1	BST1	RPS4X	RPS4X	PSMA1
GABBR2	GABBR2	C17orf62	C17orf62	CALCOCO2					CYFIP1	RPS8	RPS8	RBM14
GRID2IP	GRID2IP	CAND1		CAND1		CAND1		CAND1	DOCK2		RRBP1	RBM22
HCK		CBX3		CD109	CD109	CD109	CD109		HVCN1		SF1	RBMX
IGF2R		CD109		CD38	CD38	CD38	CD38	CD38	ITGAL	14	18	RPL10
ILK	ILK	CD38		CD44	CD44	CD44	CD44	CD44	LAMTOR1			RPL10A
KIAA1462		CD44		CD55	CD55	CD55	CD55	CD55	RAP2B			RPL18A
KPNA2	KPNA2	CD55		CHMP1A				CHMP1A	SLC2A5			RPL28
LIN7A	LIN7A	CERS6	CERS6	COPE	COPE				STOM			RPL39
LYN	LYN	CHMP1A		CORO7					STXBP2			RPL4
MAP1S	MAP1S	CNIH4						CPNE8	STXBP3			RPL9
MAP4K4		COPE	COPE	CSNK2B		CSNK2B		CSNK2B	TMEM30A			RPLP1
MEF2C	MEF2C	CORO7		CSTB		CSTB		CSTB	TMEM63A			RPS15
MMP14		CSDE1						CTNND1	TOLLIP			RPS24
MOB4	MOB4	CSNK2B		CYFIP1		CYFIP1		CYFIP1	VAMP8			RPS29
MYO5A	MYO5A	CSTB		DENND1A	DENND1A							RPS3A
NSMF	NSMF	CYFIP1		DIAPH1	DIAPH1	DIAPH1	DIAPH1					RPS4X
PHB2	PHB2	DCTN5						DNAJA2				RPS8
PI4KA		DDRGK1	DDRGK1	DOCK2		DOCK2		DOCK2				SF1
PPFIBP1	PPFIBP1	DHCR24	DHCR24					DPYSL2				SF3B2
PTPRD	PTPRD	DHRS4	DHRS4									SF3B4
RAB3GAP1	RAB3GAP1	DIAPH1		F11R				F11R				SF3B6
RAB4A	RAB4A	DNAJB11		FAM91A1								SLIRP
RAP2B		DOCK2		FKBP15								SMNDC1
RPL10A	RPL10A	EDEM1	EDEM1					GART				SND1
RPL18A	RPL18A	ERMP1	ERMP1	GIGYF2								SNRPB
RPL4	RPS3A	FAF1	FAF1	GNAS				GNAS				SNRPE
RPL9		FAM91A1		GOLGA7		GOLGA7		GOLGA7				SRRM2
RPLP1		FDFT1	FDFT1					GSTK1				TARDBP
	RPS4X	FKBP15		HCK								XPO5
RPS15		GIGYF2						HNRNPL				44
RPS29		GNAS		HVCN1	HVCN1	HVCN1	HVCN1					
RPS3A		GOLGA7		IGF2R	IGF2R	IGF2R	IGF2R	IGF2R				
RPS4X		HCK		ITCH				ITCH				
RPS8		HVCN1		ITGAL	ITGAL	ITGAL	ITGAL	ITGAL				
RRAS		IGF2R		KIAA0196								
SCIN		IKBIP	IKBIP	LAMTOR1	LAMTOR1	LAMTOR1	LAMTOR1	LAMTOR1				
SCRIB	SCRIB	ING2		LAMTOR4								
SDCBP	SDCBP	ITCH						LIN7A				
SIPA1L1	SIPA1L1	ITGAL						LRRC57				
SLC16A1	SLC16A1	KIAA0196						LUZP1				
SLC29A1	SLC29A1	KTN1	KTN1					LYN				
SLC30A1	SLC30A1	LAMTOR1		M6PR	M6PR							
SSH1	SSH1	LAMTOR4		MAGT1	MAGT1	MAGT1	MAGT1					
STAU1	STAU1	LRRC8C	LRRC8C	MEFV								
STRN	STRN	LYN		MFSD10	MFSD10							
STRN4	STRN4	M6PR		MGST1	MGST1	MGST1	MGST1					
STX11	STX11	MAGT1	MAGT1	MMP14								
STX12	STX12	MFSD10		MYD88								
STXBP2	STXBP2	MGST1	MGST1	MYO5A		MYO5A		MYO5A				
STXBP3	STXBP3	MGST3	MGST3	NCALD	NCALD							
TJP2		MMP14		NCKAP1L	NCKAP1L	NCKAP1L	NCKAP1L	NCKAP1L				
TNK2		MOB4		NECAP1	NECAP1			NT5E				
VTI1B	VTI1B	MYD88						PCYOX1				
67	45	MYO5A		PI4KA				PI4KA				
		NCALD		PML				PLSCR1				
		NCKAP1L						PSMA1				
		NECAP1		PSMA5		PSMA5		PSMA5				
		NOMO2	NOMO2					PSMC6				
		NSMF		PSMD1		PSMD1						
		NUP62						PTBP1				
		NUP98						PTPRD				
		PARP1		RAB11FIP1	RAB11FIP1							
		PCYT1A	PCYT1A					RAB3GAP1				
		PIGK	PIGK	RAB32	RAB32							
		PLP2	PLP2	RAB4A	RAB4A	RAB4A		RAB4A				
		PLSCR1		RABGEF1								
		PML	PML	RAP2B	RAP2B	RAP2B	RAP2B	RAP2B				
		POR	POR					RBMX				
		PREB	PREB									
		PSMA5										
		PSMD1		RGS19				RPL10A				
		PTDSS1	PTDSS1					RPL28				
		PWP1						RPL4				
		RAB11FIP1						RPS29				
		RAB32						RPS3A				
		RAB3GAP1	RAB3GAP1					RPS4X				
		RAB4A						RPS8				
		RABGEF1						RRAS				
		RAE1		SAMD9L								
		RANGAP1						SCIN				
		RAP2B						SCRIB				
		RGS19		SDCBP		SDCBP		SDCBP				
		RPL10						SLC16A1				
		RPL4		SLC2A5	SLC2A5	SLC2A5	SLC2A5	SLC2A5				
		RPS29	RPS29	SLIRP		SLIRP		SMC2				
		RPS3A		SND1				SND1				
		RPS8		SNX18	SNX18			SNX18				
		RRBP1	RRBP1					SRPR				
		SAMD9L						SRSF7				
		SDCBP	SDCBP					STAU1				
		SEC61A1	SEC61A1	STOM	STOM	STOM	STOM	STOM				
		SEC63	SEC63				STXBP2	STXBP2				
		SLC2A5						STXBP3				
		SLC30A1		STX11		STX11						
		SLIRP		STX12	STX12	STX12						
		SNX18		STXBP2	STXBP2	STXBP2						
		SRPR	SRPR	STXBP3		STXBP3						
		SSR1	SSR1	SURF4	SURF4	SURF4	SURF4					
		STAU1		SYK								
		STIM1	STIM1	TAP2	TAP2							
		STOM		TCIRG1	TCIRG1	TCIRG1	TCIRG1					
		STRN3		TFRC	TFRC			TFRC				
		STX11						TGFBI				
		STX12		TMEM30A	TMEM30A	TMEM30A	TMEM30A					
		STXBP2		TMEM33								
		STXBP3		TMEM63A	TMEM63A	TMEM63A	TMEM63A					
		SURF4	SURF4					TMEM109				
		TAP2	TAP2					TMEM63A				
		TBL2	TBL2	TNK2	TNK2							
		TCIRG1		TOLLIP		TOLLIP		TOLLIP				
		TECR	TECR	TPCN1								
		TFRC		VAMP8	VAMP8	VAMP8	VAMP8	VAMP8				
		TGFBI			VMA21							
		TMEM109	TMEM109	VAT1		VAT1		VAT1				
		TMEM167A		VIPAS39								
		TMEM30A		VMA21								
		TMEM33	TMEM33	VTA1				VTA1				
		TMEM50B	TMEM50B	VTI1B	VTI1B	VTI1B		VPS13D				
		TMEM63A		WDFY1								
		TNK2		WDR11								
		TOLLIP		92	43	43	23	83	16			
		TP63										
		TPCN1										
		TRIM22										
		TRRAP										
		UFL1	UFL1									
		VAMP8										
		VAT1										
		VIPAS39										
		VKORC1L1	VKORC1L1									
		VMA21	VMA21									
		VTA1										
		VTI1B	VTI1B									
		WDFY1										
		WDR11										
		XPO7										
		150	48									

Supplemental Table S2. Classification of GFP-SARS-CoV-2 E protein interactors according to GoTERM category of cellular compartments. Proteins classified into the distinct cellular region or cell compartment are listed; the total number of proteins is indicated at the bottom. Color assigned to the different categories is according to Figure 3B.

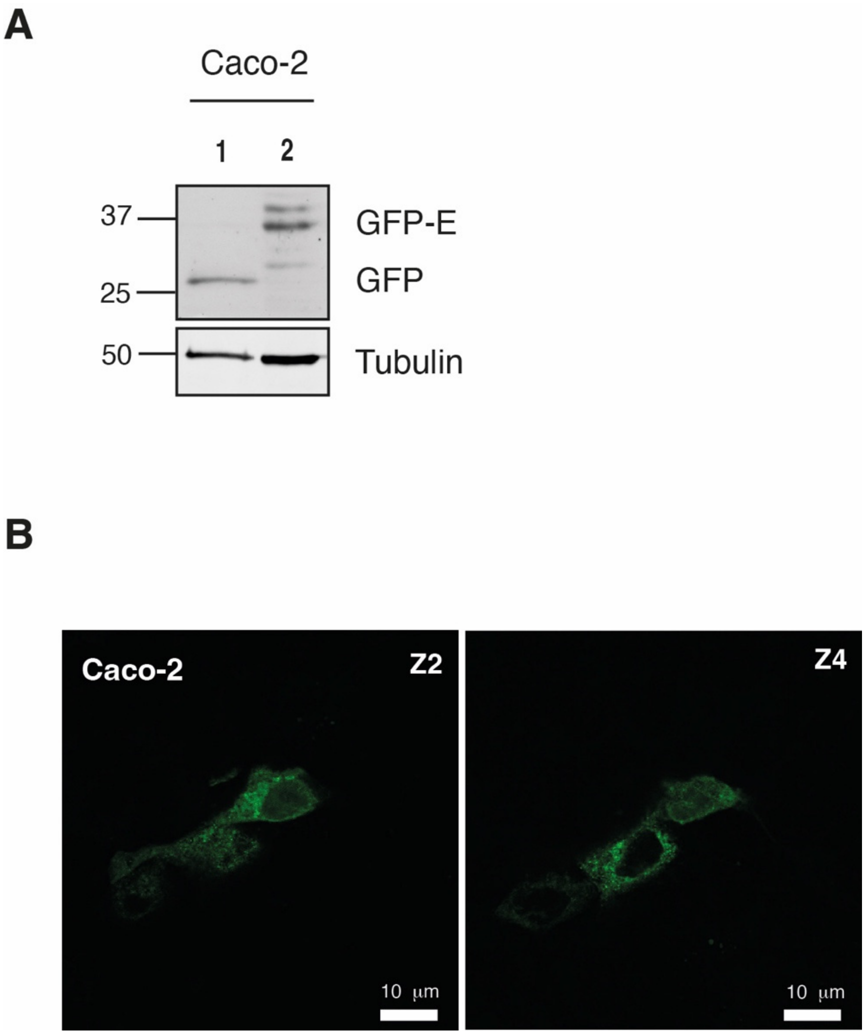

Supplemental Figure S1. Expression of the recombinant GFP SARS-CoV-2 E protein in Caco-2 cells. (A) Cells were transfected with either the plasmid pEZYeGFP (empty vector; line 1) or with the pEZYeGFP-SARS-CoV-2 E construct (line 2). After 24 h, cells were lysed and analyzed by Western Blot using anti-GFP and anti-tubulin Abs. (B) Caco-2 cells were seeded into coverslips, transfected with the pEZYeGFP-SARS-CoV-2 E construct and next day fixed and analyzed by confocal microscopy. Two different confocal planes (Z) of a representative transfection are shown.

A

Pearson´s coefficient (-1 to 1) 0.60

Li´s intensity correlation (-0.5 to 0.5) 0.28

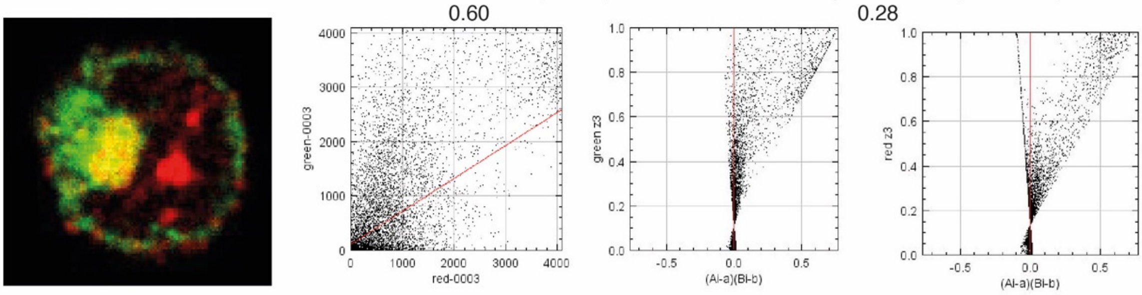

M1 coefficient: fraction of channel A (GFP-E) overlaping to channel B (syntenin): 0.609

M2 coefficient: fraction of channel B (syntenin) overlaping to channel A (GFP-E): 0.470

B

Pearson´s coefficient (-1 to 1) 0.703

Li´s intensity correlation (-0.5 to 0.5) 0.34

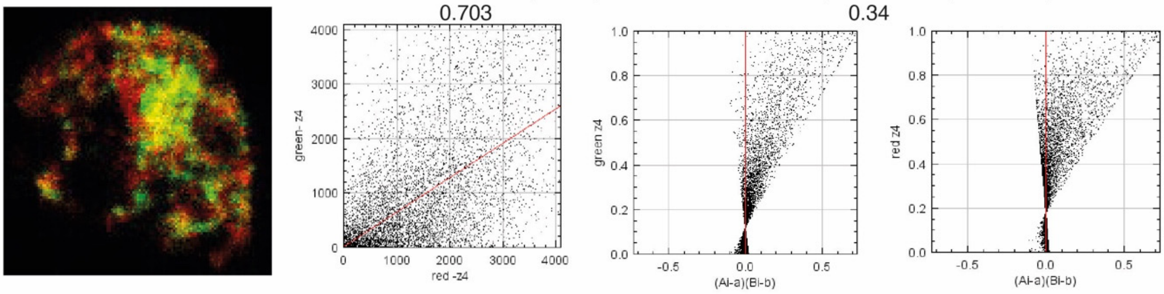

M1 coefficient: fraction of channel A (GFP-E) overlaping to channel B (ZO-2): 0.442

M2 coefficient: fraction of channel B (ZO-2) overlaping to channel A (GFP-E): 0.796

C

Pearson´s coefficient (-1 to 1) 0.713

Li´s intensity correlation (-0.5 to 0.5) 0.32

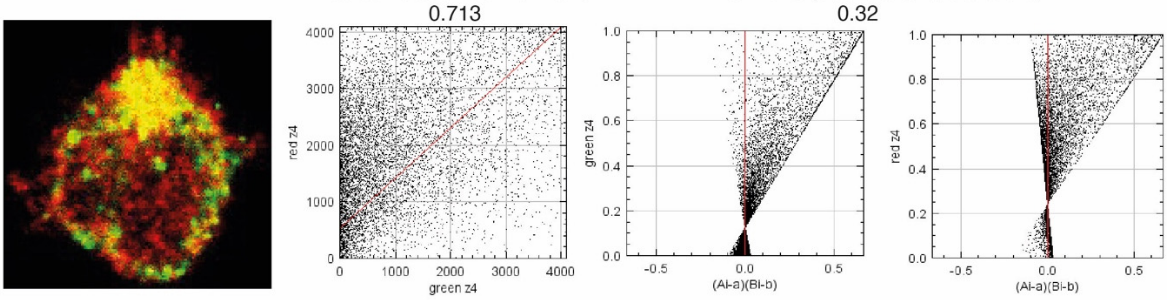

M1 coefficient: fraction of channel A (GFP-E) overlaping to channel B (IL-16): 0.650

M2 coefficient: fraction of channel B (IL-16) overlaping to channel A (GFP-E): 0.596

Supplemental Figure S2. GFP-SARS-CoV-2 E protein co-localization with PDZ proteins in THP-1 monocytes. Representative analysis of the co-localization of the GFP-SARS-CoV-2 E protein with the PDZ proteins (A) syntenin, (B) ZO-2 and (C) IL-16. The images shown were analyzed with the JaCoP plugin. Mander´s coefficient was used to determine the concurrence of both proteins, while Pearson´s coefficient and Li´s intensity correlation were used for analysis of fluorescence intensity correlation.

Mander´s coefficient (ranging from 0 to 1) was determined using automatic threshold. M1 corresponds to the fraction of channel A (green; GFP-E) that overlaps with channel B (red; PDZ protein), while M2 corresponds to the fraction of channel B (red; PDZ protein) overlapping with channel A (green; GFP-E). M values are indicated below for each image.

Pearson´s coefficient (ranging from -1 to 1) and Li´s intensity correlation (ranging from -0.5 to 0.5), were also used. In all the cases the analysis showed partial co-localization since concurrence was restricted to certain cell regions.
